# Water quality change and pollution source accounting of Licun River under long-term governance

**DOI:** 10.1038/s41598-022-06803-6

**Published:** 2022-02-17

**Authors:** Minghui Zhang, Lin Wang, Chunxia Mu, Xuda Huang

**Affiliations:** 1 College of Environmental Science and Engineering, Ocean University of China, Qingdao, 266100 China; 2 Qingdao Public Utility Construction Management Center, Qingdao, 266100 China

**Keywords:** Environmental sciences, Hydrology

## Abstract

Urbanization and human activities have exerted a tremendous adverse influence on the water quality of the Licun River, Qingdao, China. In order to restore the water quality, a succession of measures have been carried out since 1996, mainly encompassing flood controlling, sewage intercepting and watercourse greening (before 2007), watercourse and point source control based on administrative region (2008–2017), as well as the comprehensive governance based on river basin (after 2018). In 2019, the amount of discharged industrial wastewater, chemical oxygen demand, and ammonia nitrogen decreased by 53.91%, 87.75% and 89.88%, respectively, compared with 2000. Such results indicate that continuous governance has achieved a quantitative effect, and that industrial discharge is not the main pollution source. In the present work, the Spearman rank correlation coefficient and river comprehensive pollution index methods were used to analyze the change trend of main pollutants. The water quality was improved continuously, and the reduction of total phosphorus and ammonia nitrogen was the key to upgrading water quality. Afterward, the emission of pollution sources was accounted for from viewpoints of the point source, non-point source and sludge. Finally, suggestions were put forward to improve the water quality of the Licun River and provide some reference for the urban river management in northern China.

## Introduction

**Figure 1:**
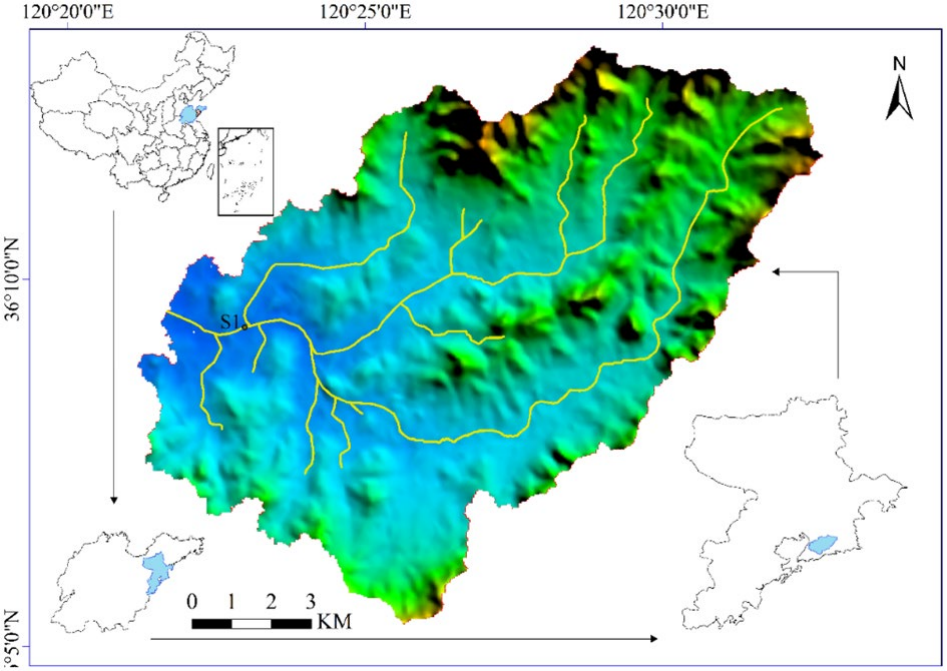
Licun River basin. Cartographic software: ArcGIS online (https://www.esri.com/en-us/home).

The Licun River is the largest river into the sea in the main urban area of Qingdao, Shandong Province, China (Fig. 1). It consists of ten main tributaries, such as the Dacun River, Zhangcun River and Shuiqinggou River. The total length of the water system is about 50 km, with the main stream of about 17 km, and the basin area is 137 km^2^. Human activities, including industrial and agricultural production, have profoundly changed the layout and hydrology of the Licun River. With the urban expansion, water conservation owing to the basin is diminished, and the surface runoff is reducing. Additionally, restricted by natural conditions, infrastructure construction, economic flourishment, and weak supervision, the Licun River has gradually become the seasonal flood channel and sewage receiving river.

In 2019, the population on the Licun River basin was added up to 1.3 × 10^6^, with 622 people per square kilometer. About 1.66 × 10^7^ t of industrial wastewater and 3.11 × 10^5^ t of domestic sewage were discharged per day. With the booming economy and soaring population, although many governance measures have been conducted over the past 20 years, the water quality pollution still rose above an acceptable level for a long time. What is worse, in 2016, three sections of Licun River were listed as black and odorous water by the Ministry of Housing and Urban–rural Development and the Ministry of Ecology and Environment. This suggested that previous governance schemes in improving the water quality of the Licun River have not worked effectively^1–3^.

Based on the above, by combing the past governance focus and water quality changes, the water quality changes caused by the change of governance focus are intuitively displayed. Moreover, according to the long-time changes in water quality, the main pollutants during change are identified to avoid the identification error induced by the short-time accidental mutation of water quality. Additionally, the quantitative changes in pollutant discharge and water quality under continuous governance are analyzed using the river comprehensive pollution index and Spearman rank correlation coefficient methods. Finally, based on the calculation of pollution sources in the basin, the main pollutants production is clarified, and suggestions are put forward for pollution control, which will provide a reference value for the future governance of the Licun River and other rivers in northern China.

## Materials and methods

### Field sampling

Water samples were collected from the Shengli Bridge section (the only national monitoring site in the basin), and marked as S1, as shown in Fig. 1. Due to the seasonal cutoff of the middle and upper reaches of the river, the monitoring data of S1 were used to analyze the long-term water quality change. Samples were collected twice a week from January 2018 to December 2020. Historical water quality data were collected from Qingdao Environmental Protection Bureau and Qingdao Environmental Status Bulletin. The above sampling and determination methods strictly conform to relevant surface water monitoring technical specifications and Chinese standard methods^4,5^.

### Data analysis

The water quality was monitored and analyzed according to GB3838-2002 and evaluated adopting the river comprehensive pollution index method^6^. According to the current water quality of the Licun River Basin and the exceeding standard items of previous water quality, five pollutant indicators were selected as evaluation indicators, i.e., 5-day biochemical oxygen demand (BOD_5_), ammonia nitrogen (NH_3_-N), petroleum, total phosphorus (TP) and chemical oxygen demand (COD). Spearman rank correlation coefficient method is widely^7^ used to measure the statistical significance of time series change trend, especially in the study of change trend of pollutant concentration^8^. This method can help to test the correlation between the index data series and response time series of water quality so as to determine the trend change in the water quality sequence in the time series.

The value of *r*_*s*_ (Spearman rank correlation coefficient) was estimated by Eqs. (1) and (2). Where *X*_*i*_ and *Y*_*i*_ are the sequence numbers of the period 1 − *N* in descending order of concentration and time, and *N* is 21 (the number of items participating in the evaluation of pollution indicators).

1 $$ r_{s} = 1 - \frac{{6\mathop \sum \nolimits_{i = 1}^{N} d_{i}^{2} }}{{N^{3} - N}}, $$

2 $$ d_{i} = X_{i} - Y_{i}. $$

The absolute value *r*_*s*_ was compared with the critical value (*W*_*p*_ = 0.435/0.556) in Supplementary Table S1. When *r*_*s*_ is greater than *W*_*p*_, the change trend is significant. Negative *r*_*s*_ indicates that index changes show a downward or upward trend in the evaluation period, while positive *r*_*s*_ implies that the index changes tend to increase in the evaluation period. When *r*_*s*_ is no more than *W*_*p*_, the change trend is insignificant, and the water quality is stable during the evaluation period.

The value of *P* (river comprehensive pollution index) and *K* (pollution sharing rate) were estimated by Eqs. (3)–(5). Where *C*_*i*_ is the measured value of the *i*th parameter, *C*_*0*_ is the standard value of the *i*th parameter, and n is the number of parameters. In this paper, *C*_0(DO)_ = 2 mg/L, *C*_0(COD)_ = 40 mg/L, *C*_0(NH3-N)_ = 2 mg/L, *C*_0(petroleum)_ = 1 mg/L, *C*_0(TP)_ = 0.4 mg/L, and *C*_0(BOD5)_ = 10 mg/L.

3 $$ P = \frac{1}{n}\mathop \sum \limits_{i = 1}^{n} P_{i} , $$

4 $$ P_{i} = \frac{{C_{i} }}{{C_{0} }}, $$

5 $$ K_{i} (\% ) = \frac{{P_{i} }}{P} \times 100. $$

*P* ≤ 0.8 means the water is qualified and basically meets the corresponding functional standards, with only some indicators exceeding the standard (within one time). 0.8 < *P* ≤ 1.0 means the water is basically qualified, with a few water quality indicators over corresponding standards; the water function has not been significantly damaged. 1.0 < *P* ≤ 2.0 refers to water pollution, indicating limited water function, with most water quality indexes beyond corresponding standards. *P* > 2.0 denotes severe pollution; that is, the water function has been seriously damaged, with each water quality index out of the standard by more than one time on average, some even several times^7^.

The source pollution emissions^9^ from residents are calculated, as shown in Eq. (6), where *Q* denotes the output of pollutants (t), *M* means the permanent resident population, and *ε* is the intensity of pollutant generation. In Qingdao, *ε*_COD_ is 40.24 g/person/day, *ε*_NH3-N_ is 2.45 g/person/day, and *ε*_TP_ is 0.2 g/person/day.

6 $$ Q = \frac{M \times \varepsilon \times 365 }{{100}}. $$

The non-point source pollutant emission^10^ caused by rainfall is calculated in Eq. (7), where *Q* denotes the emission of non-point source pollutants, *R* represents the annual precipitation, *A*_*i*_ is the area of the *i*th underlying surface type, *ψ*_*i*_ is the runoff coefficient of the *i*th underlying surface type and *EMC*_*i*_ is the pollutant load of rainfall runoff of the *i*th underlying surface type.

7 $$ Q = \sum R \times A_{i} \times \varphi_{i} \times EMC_{i} . $$

ArcGIS Online software is used for map making. See https://www.esri.com/en-us/home for details.

## Governances course of the Licun River

The main governance measures of Licun River in different stages are described in this section. Obviously, the governance pivot is transferred from sewage collection and channel regulation to the management of watercourse and point source based on administrative region, and from flood protection and sewage interception to the comprehensive control. The constant adjustment of the Licun River governance system to cater to urban development brings about advantages and serious challenges. Accessible facilities like dikes and dams and measures like river hardening have caused hydroelasticity loss in the river system^11,12^. In addition, with economic prosperity and increasing population density, the potential damage caused by future floods and water pollution is also increasing^13,14^.

### Changes in sewerage collection and treatment in the Licun River basin

As of 2016, the density of the drainage network is about 17.62 km/km^2^, and the average collection rate of sewage is over 97%. Three sewage treatment plants are located in the Licun River basin, namely, the Licun River Sewage Treatment Plant, the Zhangcun River Water Purification Plant and the World Horticultural Exposition Reclaimed Water Purification Plant (Supplementary Fig. S1). The historical overview of sewage treatment plant construction is shown in Table 1.

**Table 1:** Construction history of sewage treatment plants in the Licun River basin.

Sewage treatment plant	Historical construction
Licun River sewage treatment plant	(1997–2008) Processing capacity: 80,000 t/day; Effluent quality: the Class II standard of GB8978-1996; (2008–2011) Processing capacity: 170,000 t/day; Effluent quality: the Class II standard of GB18918-2002; (2011–2016) Effluent quality: Class A of the first-level standard of GB18918-2002; (2016–2019) Processing capacity: 250,000 t/day; (2019–2020) Processing capacity: 300,000 t/day; Effluent quality: the Class IV standard of GB3838-2002(COD, NH_3_-N and TP), the Class V standard of GB3838-2002 and Class A of the first-level standard of GB18918-2002 (other)
Zhangcun River water purification plant	(2018–2020) Processing capacity: 40,000 t/day; Effluent quality: Class V of GB3838-2002
World horticultural exposition reclaimed water purification plant	(2014–2020) Processing capacity: 6000 t/day; Effluent quality: Class A of the first-level standard of GB18918-2002

### The history stages of river governance

**Figure 2:**
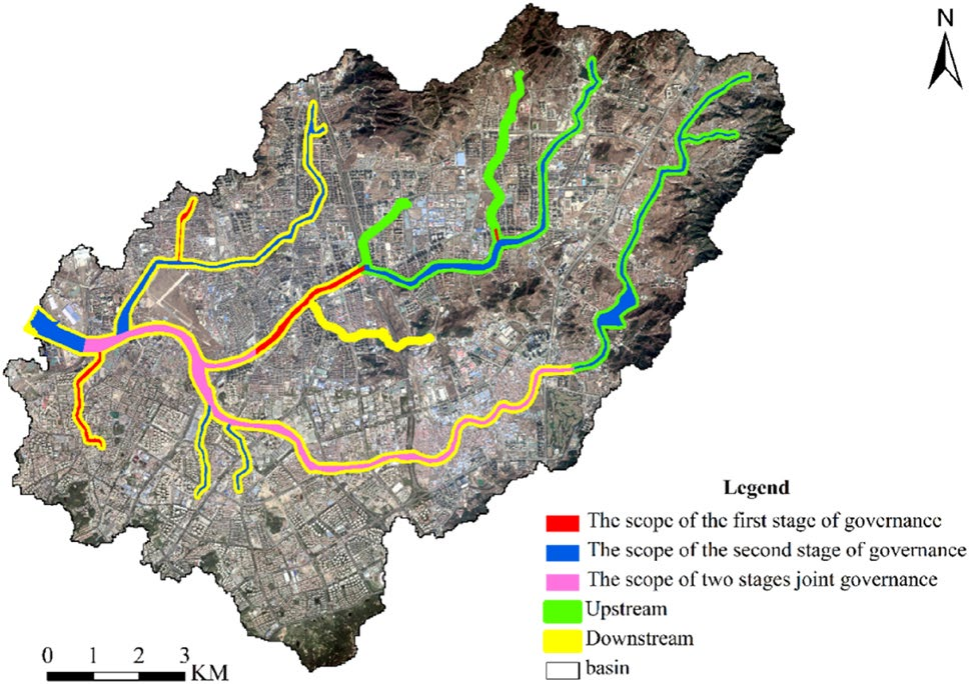
Schematic diagram of governance scope in the first and second stages. Cartographic software: ArcGIS online (https://www.esri.com/en-us/home).

According to the differential governance focuses and methods, the governance of the Licun River Basin was divided into three stages. The governance scope in the first and second stages is marked in Fig. 2.

#### The first stage of governance (2000–2007)

In this stage, the river governance focuses on the watercourse by resorting to greening, flood control and sewage interception, as shown in Table 2.

**Table 2:** Overview of the first governance stage.

Object	Governance situation
Licun River	(2001) Dredging, greening and laying of sewers; (2006) dredging and left bank revetment
Zhangcun River	(2002–2003) Greening, flood control (50-year return period), pollution interception; (2006) rainwater pipelines construction
Other tributaries	(2001) Flood control: Jinshui River (20-year return period), Xiliuzhuang River (10-year return period), Xiaowengcun River (10-year return period), Shuiqinggou River (10-year return period)

The dirty water of poor quality in the Licun River had been initially managed in this stage. Although a lot of money had been invested in pollution control, the water quality still failed to achieve the expected effect, and not to mention the pollution problem. Such results were attributed to the long-time pollution accumulation and the limited development, governance mode and technical conditions.

#### The second stage of governance (2008–2017)

In the second stage, the governance focus was shifted to the watercourse and point source based on the administrative region and upstream and downstream, as is detailed in Table 3.

**Table 3:** Overview of the second governance stage.

River	Reach	
	Upper reaches	Lower reaches
Licun River	(2009–2010) Pollution interception, greening, pipeline construction, flood control; (2013) flood control (50-year return period)	Flood control, waterside space construction (wetland, water fall, dam), sewage interception, reconstruction of main pipes, rainwater engineering, river bank restoration and reconstruction (multi-natural ecological revetment), desilting
Zhangcun River	Bank protection	(2011) Water replenishment engineering (Tsingtao Brewery No.2 Factory; Class A of the first-level standard of GB18918-2002; 6000-7000t/day); (2014) dredge, revetment, flood control, waterside space construction (wetland, water fall, dam), river bank restoration and reconstruction (multi-natural ecological revetment), Sewage interception, reconstruction of main sewage pipes, rainwater pipelines construction
Other tributaries	(2013) flood control: Yangjiaqun River; Dacun River (50-year return period)	(2006) Dacun River: flood control (20-year return period); (2009) Hexi River: sewage interception, bank protection, water storage, greening; (2008) Yangjiaqun River: sewage interception, bank protection, water storage, greening and flood control (20-year return period)

After adopting the above measures, the water quality of the upper reaches was significantly improved, and the water quality of the monitoring section was improved continuously. However, black and odorous water appeared in some areas downstream, accompanied by serious pollution problems^15^.

#### The third stage of governance (2018–2020)

In the third stage, new ideas for pollution control of the Licun River were proposed, encompassing improvement, cleaning, diversion, restoration and governance^16^. This stage advocated that pollutants are reduced from the source, and technology is embraced to improve pollutant discharge standards. The ecological restoration is promoted while management and maintenance are srengthened^17^. As the environmental capacity is enlarged, the possibility of pollution will be reduced, and the pressure caused by river basin pollution will be released more^18,19^.

##### Improvement (sewage treatment capacity and effluent quality^20^)

The Licun River Sewage Treatment Plant has presented improved treatment capacity (300,000 t/day) and effect since 2019. The effluent COD, NH_3_-N and TP meet the Class IV standard of GB3838-2002, while the other indicators meet the Class V standard of GB3838-2002 and the Class A standard of GB18918-2002. Also, the Zhangcun River Water Purification Plant has shown increasing treatment capacity (40,000 t/day) and effect since 2018, with the effluent meeting the Class V standard of GB3838-2002.

##### Cleaning up (silt and point sources^20^)

A total of 60 × 10^4^ m^3^ of siltation was cleared in the Licun River basin. Moreover, the discharge of 384 point sources along the river was treated. Moreover, temporary sewage interception facilities were removed, and the pipe network system was upgraded. Enterprises failing to meet the discharge standards should ensure that most sewage is drained off the pipe network.

##### Diversion (separate rainwater from sewage^20^)

Stormwater lines were constructed independent of sewer lines, and for 23 rural communities along the river, rainwater and sewage were separated. In order to prevent sewage from being mixed into the rainwater pipe network, the damaged drainage pipes were repaired, and partial low-flow sewage pipes were replaced.

##### Restoration (water replenishment and ecological restoration^20^)

The construction of water supply projects was strengthened, and water supply pipelines were installed. Wuyang Road (50,000 t/day) and Sanjiaodi Water Supply Points (150,000 t/day) had been constructed to prompt water supply. In order to restore the self-purification of the river, wetlands were developed, where aquatic plants were planted.

##### Governance (governance responsibility system and intelligent water quality monitoring system^20^)

The governance responsibility system and the river governance and maintenance assessment system were established. The watercourses were divided into sections with community units, and 94 managers were set for gridding and refined governance. The joint review mechanism of drainage permits and the coordination and dispatching mechanism of point source investigation were established. A list of water-related industrial enterprises was generated. The Intelligent water quality monitoring system was built at the river confluence, tributary estuaries and main drainage outlets.

## Analysis of improvement effect of governance

### Quantitative effects of continuous governance

Supplementary Figure S2 shows that the discharge of industrial wastewater, COD and NH_3_-N has steadily declined over the past 20 years. Compared with those in 2002, the discharge of industrial wastewater, COD and NH_3_-N decreased by 53.91%, 89.88% and 87.75% in 2019, respectively. In fact, with the relocation of most industrial enterprises and the improvement of the pipe network, the industrial waste water that meets the discharge standards has gone into the pipes and does not directly enter the river to cause pollution. Taken together, these results seemed to indicating that domestic sewage and the non-point source pollution had become the main pollution sources of the Licun River. The highest decline rate (compared to the previous year) of NH_3_-N could be found in 2009, about 55.2%, while the highest decline rate of COD emission was 71.3% in 2016. These results indicated that the basin governance, the transformation and upgrading of old industrial areas, and other control measures in the first and second stages had achieved remarkable results. However, with the implementation of pollution control, pollutant emission did not decline continually. For example, the emissions of COD and NH_3_-N were both rebounded. Therefore, the coordinated development of the environment and economy will not be realized naturally but will rely on people’s active efforts^21^.

### The qualitative change effect of continuous governance

The effect of water pollution control was reflected by the changing concentrations of main pollutants. Based on the monitoring data of the Licun River from 2000 to 2020, the change trends of main pollutants and water quality were analyzed and evaluated using the comprehensive pollution index and Spearman rank correlation coefficient methods^22,23^. The main pollutants were identified, and then the main pollutant emissions of different pollution sources were calculated. Based on this, reasonable suggestions were initiated to stabilize and improve the water quality of the basin.

The annual average monitoring data were quantitatively analyzed by the Spearman rank correlation coefficient method (Table 4).

**Table 4:** Rank correlation coefficients of major pollutants (2000–2020).

Section	Absolute value of *r*_*s*_					
	*r*_*s* (DO)_	*r*_*s* (COD)_	*r*_*s* (NH3-N)_	*r*_*s* (petroleum)_	*r*_*s* (TP)_	*r*_*s* (BOD5)_
S1	0.883 (> 0.435)	0.861 (> 0.435)	0.838 (> 0.435)	0.787 (> 0.435)	0.865 (> 0.435)	0.843 (> 0.435)

As shown in Table 4, the above absolute values are all higher than W_P_, indicating a significant change trend. Among them, *r*_*s*(DO)_ is positive, implying that DO is rising during evaluation. In contrast, other *r*_*s*_ values are negative, revealing that the changes of indexes are in a downward trend during evaluation. To sum up, these trends show the improving water quality in the Licun River basin^24^.

**Figure 3:**
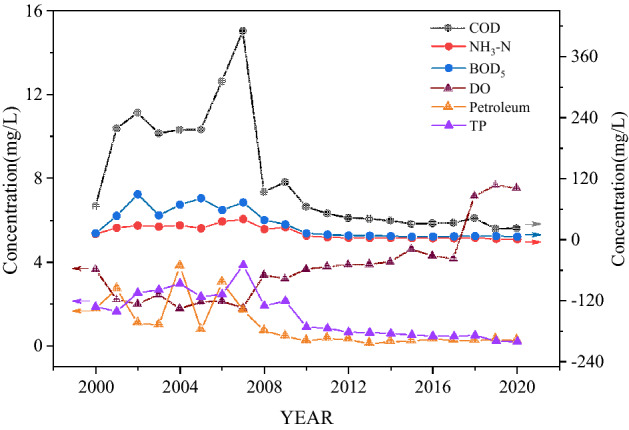
The annual average concentration of main water quality indicators at S1.

**Figure 4:**
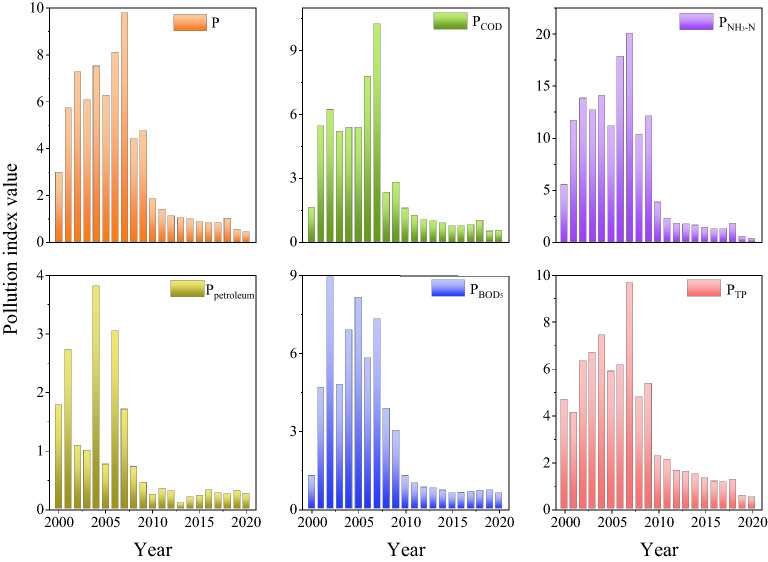
Change trend of pollution index.

From 2000 to 2020, the annual average concentrations of main water quality indicators in the monitoring section are shown in Fig. 3. Before 2010, the annual average concentration of main pollutants fluctuated significantly. Despite some measures, the main pollutants still presented an obvious growth in concentrations, showing that flood control and greening measures in the early stage did not significantly improve water quality. Moreover, the concentrations fluctuated greatly due to the improvement of the discharge standard and the lack of pollution supervision. After 2010, main pollutants showed a steady downward trend in the annual average concentration, while the concentration of dissolved oxygen (DO) increased significantly. This indicated the positive role of governance measures in improving water quality at this stage. On this basis, the comprehensive pollution index method was used to calculate the single and comprehensive pollutant indexes (Fig. 4).

Figure 4 shows that the change trend of comprehensive pollution index (P) of main pollutants rises first and then decreases, reaching the peak value (9.79) in 2007, basically consistent with the change trend of the proportion of GDP of the secondary industry (especially industrial GDP) in the total GDP. According to the survey results of industrial structure in the Licun River Basin, the proportions of both the secondary industry GDP and industrial GDP in total GDP reach the peak in 2007 (52.43% and 47.91%, respectively). The comprehensive pollution index drops sharply in 2008, indicating that the governance measures in response to point source control in this stage have achieved a short-term rapid improvement in water quality. The comprehensive pollution index rises slightly in 2009, then decreases steadily, and finally remains stable, indicating that the governance at this stage presses ahead with the water quality control but is still not enough. The slight rebound of the comprehensive pollution index in 2009 also demonstrates the importance of supervision and long-term governance after measures implementation. After 2018, the comprehensive pollution index decreases significantly, indicating that the new stage of governance measures come into force again.

**Figure 5:**
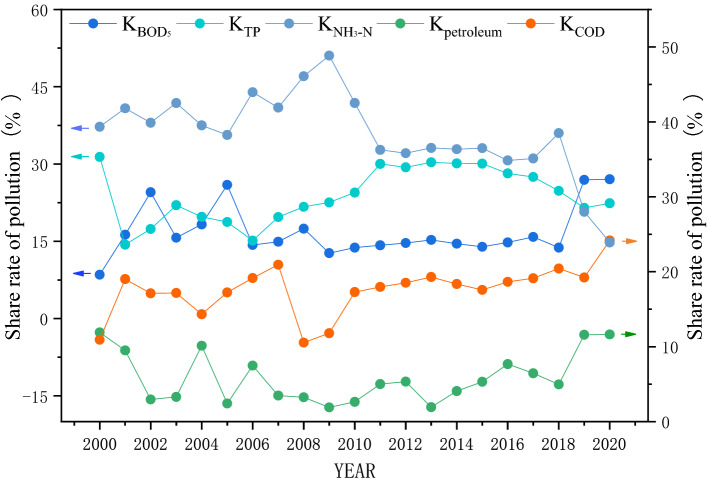
Pollution contribution rate of major pollutants (2000–2020).

The change trend of each single pollution index (*P*_COD_, *P*_NH3-N_, *P*_petroleum_, *P*_BOD5_, and *P*_TP_) was consistent with that of the comprehensive pollution index (*P*). The peak values of *P*_petroleum_ (3.81) and *P*_BOD5_ (8.92) are in 2004 and 2003, respectively, indicating that the river was greatly affected by the coastal industrial pollution in 2004. This could be attributed to the poor effect of governance on industrial sewage, the low discharge standard, and illegal discharge into the river. With the increasing requirements for environmental protection and upgrading governance means, the organic pollutants in rivers gradually decreased owing to the sound emission standards and governance of point source pollution. After 2009, the pollution index decreases significantly, indicating that industrial pollution control and point source control achieve good results. In order to clarify the impact of the above major pollutants on water quality, the pollution contribution rates of each pollutant were calculated (Fig. 5).

Figure 5 shows the significant change of pollution contribution rate (*K*) of main pollutants in the Licun River Basin from 2000 to 2020, indicating the pollution has been gradually controlled. *K*_NH3-N_ represents the most significant change, increasing first and then decreasing. Before 2009, K_NH3-N_ is greater than 35% and reaches the maximum in 2009 (51%), implying serious NH_3_-N pollution. After 2009, K_NH3-N_ decreases year by year, only 14.7% in 2020, indicating seriously NH_3_-N pollution. However, the monitoring concentration still exceeds the standard occasionally, which is still worthy of attention in the future. The change trend of *K*_TP_ first changes in inverted N shape and then remains stable at about 25%. Monitoring data show that TP concentration occasionally exceeds the standard, which should be paid more attention. K_COD_ remains stable at about 17% with little fluctuation and increases to 24.2% in 2020, while the monitoring data show that COD concentration is stable and up to the standard. The standard of COD concentration is 4–100 times higher than that of other pollutants. Therefore, a slight change in COD concentration can also tremendously affect the share rate of other pollutants. Combined with the current COD concentration reaching the standard for a long time, it could be concluded that although *K*_COD_ increased slightly in 2020, it was not the main pollutant. The change trend of *K*_BOD5_ was consistent with that of *K*_COD_, which can be analyzed equally. The change trend of *K*_petroleum_ showed stable fluctuations before 2018, while an obvious increase in 2019 and 2020. The monitoring data show that the concentrations of petroleum pollutants reach the standard steadily after 2008, and the slight increase of *K*_petroleum_ in 2020 is caused by the sharp decrease of *K*_COD_ and *K*_BOD5_. Therefore, petroleum was not considered the main pollutant for the current water quality. In 2020, the contribution rate of main pollutants in water quality from large to low is *K*_BOD5_, *K*_COD_, *K*_TP_, *K*_NH3-N_ and *K*_petroleum_, indicating that the Licun River Basin was still a typical domestic polluted river, and TP and NH_3_-N reduction was the key to improve water quality in the future.

## Emission accounting of pollution sources and suggestions for control

The pollution sources of the Licun River include point source, non-point source and internal source^25^. According to the conclusion that TP and NH_3_-N are the main pollutants in the future, the pollution load of TP and NH_3_-N is calculated from the above aspects.

### Point source pollution

At present, incomplete interception of point source pollution still can be found, and domestic sewage is discharged into the main river along rainwater pipes and tributaries. A total of 11 point sources were found in the upper reaches of the Zhangcun River, a tributary of the Licun River. These point sources were all concentrated emissions from the living sources of residents. The calculation results of Eq. (6) are shown in Table 5. The COD, NH_3_-N and TP emissions of point source pollution in the Licun River Basin are 118.91 t/a, 3.57 t/a and 2.14 t/a, respectively.

**Table 5:** Discharge of point source pollutants in the river basin.

Location	Resident population (person)	Number of sewage outlets (pcs)	Sewage quantity (m^3^/day)	Emission of pollutants (t/a)		
				COD	NH_3_-N	TP
Yukuang community	1419	3	129	20.62	0.62	0.37
Hongyuan community	1781	2	141	22.54	0.68	0.41
Dongchen community	2109	2	178	28.45	0.85	0.51
Gouya community	2454	3	201	32.13	0.96	0.58
Beilongkou community	1000	1	95	15.18	0.46	0.27
Total	8763	11	744	118.91	3.57	2.14

### Non-point source pollution

With the further control of point source pollution, the impact of non-point source pollution will be gradually amplified^26^. The non-point source pollution in the Licun River basin is mainly derived from the initial rainwater. The pollution load of the initial rainwater is very high and even the concentration of the initial rainwater can exceed that of the sewage in some areas. At present, no effective initial rainwater collection and treatment system is accessible in the Licun River basin, causing the initial rainwater directly to be discharged into the river. The urban underlying surface is covered by concrete, which prohibits rainwater infiltration compared with other land use types such as forest land and grassland. For this reason, it is easier to form surface runoff in a short time, aggravating pollution by carrying more pollutants into the river.

**Figure 6:**
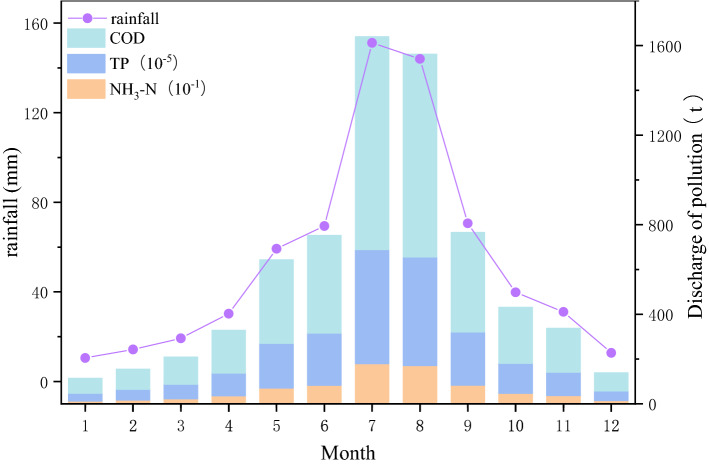
Monthly variation of non-point source pollution with rainfall in the Licun River basin.

According to the field investigation of rainwater quality in Qingdao and the range of runoff pollution load values of different underlying surfaces in literature^27^, the reference values of runoff coefficient, the area of rainwater collection in the drainage system and pollution load of different underlying surfaces in the basin were determined (Supplementary Table S2). As calculated by Eq. (7), the pollution productions of COD, NH_3_-N and TP in each month are shown in Fig. 6. Finally, the annual pollution productions of COD, NH_3_-N and TP are 4101.44t/a, 77.62t/a and 0.02t/a, respectively. In order to reduce the impact of non-point source pollution on water quality, it is necessary to take initial measures for rainwater collection and treatment.

### Sediment contamination release

With the sound control of other pollutants, the negative impact of sludge on water quality is increasingly obvious. There is a dynamic balance between absorption and release of the pollutants, sludge and river water. When the pollutant content in the river decreases, the pollutants in the sludge will be further released into water, resulting in the secondary pollution of water quality. Therefore, a scientific dredging method should be adopted to reduce TP, NH_3_-N and other pollutants by investigating the depth and state of sludge. At present, the main silted watercourses distribute in the upper reaches of the Licun River. The release rates of COD, NH_3_-N, and TP from sludge are 15, 8, and 5 mg/m^2^/day, respectively^28^. The sedimentation parameters and pollutant release of each reach are shown in Table 6. The total emissions of COD, NH_3_-N and TP in the sediments are 8.04 t/a, 4.29 t/a and 2.68 t/a, respectively.

**Table 6:** Release of endogenous pollutants in the Licun River basin.

River	Average width of main silted reach (m)	Siltation length (m)	Endogenous pollution emissions (t/a)		
			COD	NH_3_-N	TP
Licun River	100.09	7500	4.11	2.19	1.37
Zhangcun River	70	5500	2.11	1.12	0.70
Dacun River	40	6650	1.46	0.78	0.49
Houjiazhuang River	15	620	0.05	0.03	0.02
Jinshui River	20	2300	0.25	0.13	0.08
Hexi River	24	450	0.06	0.03	0.02
Total	–	23,020	8.04	4.29	2.68

### Pollution control recommendations

#### Expansion of sewage treatment plants and effluent quality enhancement

Compared with that in 2000, the emission of domestic sewage reached 3.13 × 10^5^ t in 2019, an increase of 63.84% (Supplementary Fig. S3). Moreover, the shortage rate of treatment capacity decreased from 58.17 in 2000 to 5.54% in 2019, and the sewage treatment capacity still failed to keep abreast with the increase in sewage volume. The lack of a drainage system and pipe network easily caused untreated domestic sewage to flow into the river. The investigation showed that the Licun River Sewage Treatment Plant was not expected to be expanded due to insufficient land space. Moreover, the plant is adjacent to the end of the basin; thus, the treated water directly flew into the sea without effective utilization. Therefore, it is suggested to build new sewage treatment plants in the middle or upper reaches of the basin so that high-quality treated water can be directly discharged and replenish the river. In this way, more point source emissions can be accommodated, and increased water volume will promote river self-purification, dilute pollution and reduce the residence time of pollution, thus reducing the impact of pollutants released from the sediment.

#### Replenishment of rivers with water

Licun River is a typical seasonal and intermittent river, accompanied by a large annual variation of precipitation with uneven distribution. The annual average precipitation is 662.1 mm. The annual rainfall in spring, summer, autumn and winter accounts for 17%, 57%, 21% and 5%, respectively. According to the flow statistics of the Licun River Monitoring Station over the years, the spring and winter flow is basically zero. Little precipitation can lead to the retention of pollutants and the increase of pollutant concentration. According to the current research on the relationship between flow velocity and water bloom phenomenon in China^29^, the flow velocity greater than 0.2 m/s can effectively prevent water eutrophication. Taking into account the habitat of aquatic organisms, the reasonable flow rate should be controlled at about 0.3 m/s^30^. However, the Licun River, with the current situation, fails to meet this requirement. According to the ecological water demand of the Licun River calculated by Zhang^16^ using R2-CROSS method, at least 26 × 10^4^ m^3^/day of water supply is needed. Based on the current water shortage situation in Qingdao, the treated water from the sewage treatment plant is the most resource-efficient source for water replenishment.

#### Non-point source pollution control and reasonable ecological restoration

With the management of point source pollution, the influence of urban non-point source pollution will be prominent. The control of non-point source pollution is based on long-term and perfect monitoring data of water quality and runoff^31^, thus needing finer grid monitoring. Targeted control is carried out on the basis of identifying the key source areas of non-point source pollution.

According to the field investigation, the wetland plants in the Shuiqinggou River and the lower reaches of the Licun River are mainly *aquatic iris, Lythrum* and *yellow calamus*; the wetland plants in the middle reaches are mainly *calamus, ryegrass* and *reed*. The wetland plants in the Zhangcun River Basin are mainly *calamus, red polygonum, shallot, rushes, canna, Pennisetum*, etc*.* Ecological restoration and plant planting should be linked with the actual structure of regional plant species so as to conduct reasonable wetland plant protection and planting. While purifying water quality, self-purification capacity of rivers will be improved.

## Conclusions

According to the governance focus in different periods, the consecutive governance in the past 20 years can be divided into three stages. The first stage of river engineering governance encompasses flood control, sewage interception and greening (before 2007); the second stage of river engineering and point source treatment is concentrated in the administrative region (2008–2017); the third stage of comprehensive treatment is based on the basin (after 2018). The decrease of industrial wastewater and pollutant discharge and the increase of domestic sewage collection suggest the positive effect brought by continuous governance. By adopting the Spearman rank correlation coefficient and the river comprehensive pollution index methods, the analysis results show that water quality begins to be improved with further treatment. In addition, it is concluded that industrial discharge is not the main source of pollution. Instead, the key lies in reducing TP and NH_3_-N to improve water quality in the future. The current pollution emissions of the point source, non-point source and sediment source are calculated. On this basis, suggestions for pollution control are put forward, including the construction of sewage treatment plants, replenishment of water sources and non-point source pollution control.

## Supplementary Information

Supplementary Information.

## Data Availability

The data that support the findings of this study are available from [Qingdao Ecological Environment Bureau] but restrictions apply to the availability of these data, which were used under license for the current study, and so are not publicly available. Data are however available from the authors upon reasonable request and with permission of [Qingdao Ecological Environment Bureau].
