# Supplementary material for: Water quality change and pollution source accounting of Licun River under long-term governance
Source: Sci Rep. 2022 Feb 17;12:2779. doi: 10.1038/s41598-022-06803-6 (PMC8854410; doi:10.1038/s41598-022-06803-6)
Supplement: Supplementary file 1 — Supplementary Information. [file 41598_2022_6803_MOESM1_ESM.pdf]

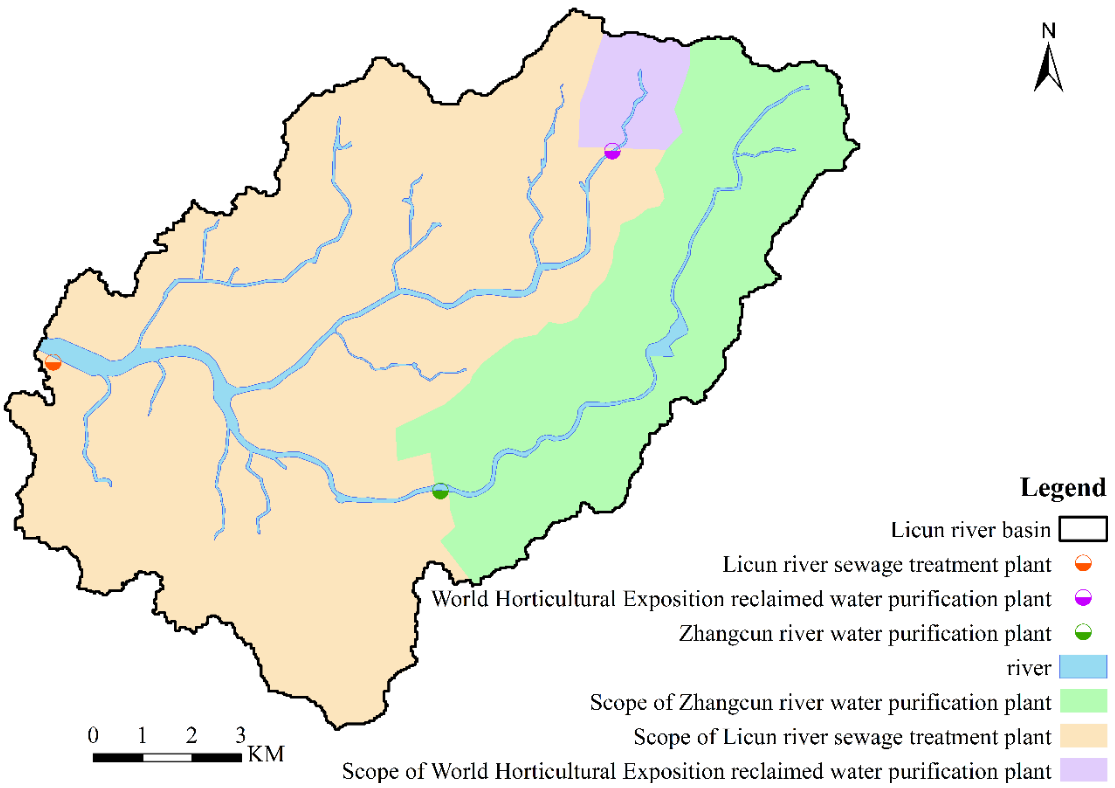

Fig. S1 Layout of sewage treatment facilities in Licun River watershed.

Cartographic software: ArcGIS online (https://www.esri.com/en-us/home)

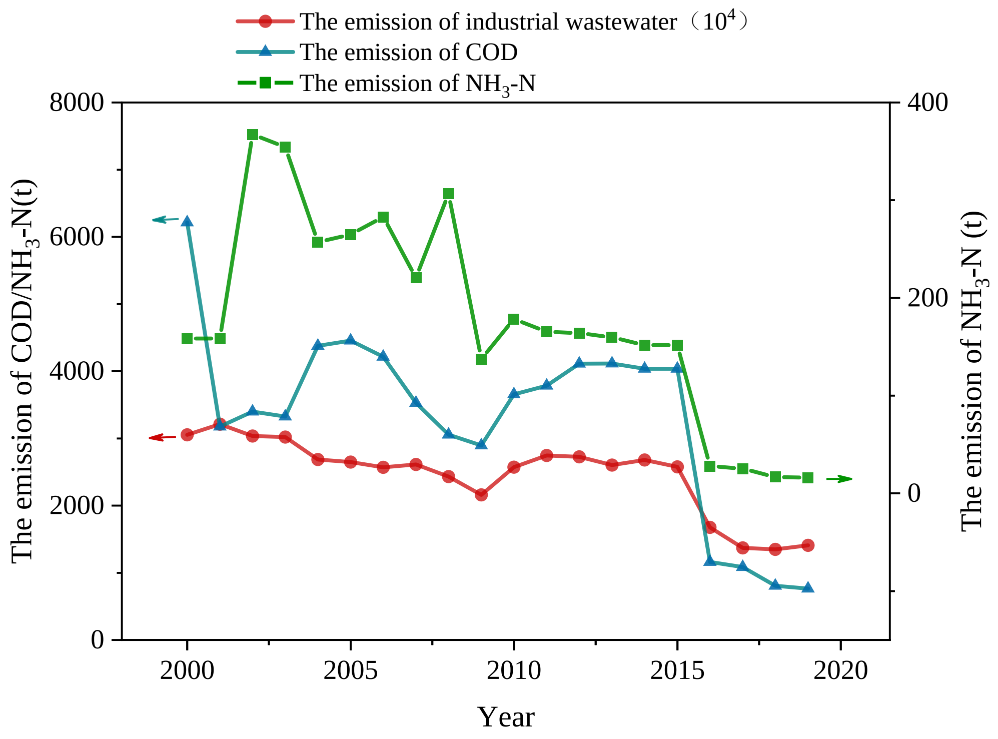

Fig. S2 Discharge of industrial wastewater, COD and NH_3-N in the Licun River Basin (2000-2019).

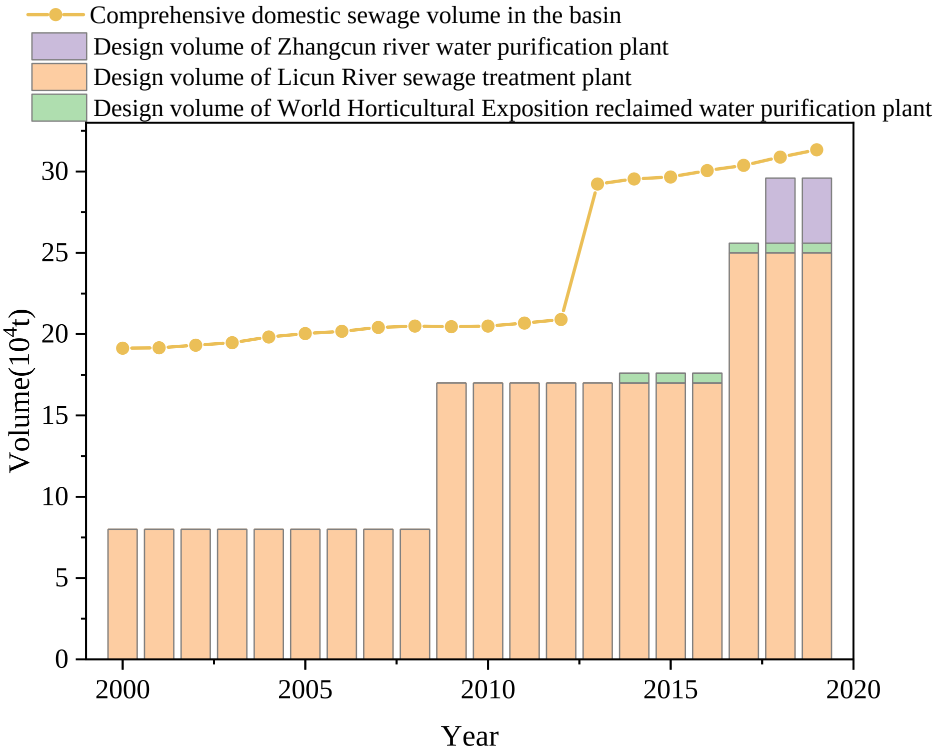

Fig. S3 Emissions of domestic sewage and sewage treatment capacity in the Licun River Basin (2000-2019).

Table S1 Comparison of critical values of rank correlation coefficient test.

N	W_P	
	Significance level (one-sided test)0.05	Significance level (one-sided test) 0.1
5	0.900	1.000
6	0.829	0.943
…	…	…
20	0.447	0.570
21	0.435	0.556
22	0.425	0.544

Table S2 Reference value of pollutant load, area and runoff coefficient.

Underlying surface type	COD (mg/L)	NH_3-N (mg/L)	TP (mg/L)	Area(km^2)	φ_i
Pavement	200	1.66	0.21	30.32	0.9

Underlying surface type	COD (mg/L)	NH_3-N (mg/L)	TP (mg/L)	Area(km^2)	φ_i
Green space	36	0.43	0.20	15.73	0.15
Roof	40	0.55	0.12	21.83	0.85
